# Functional molecular evolution of a GTP sensing kinase: PI5P4Kβ

**DOI:** 10.1111/febs.16763

**Published:** 2023-03-10

**Authors:** Koh Takeuchi, Miki Senda, Yoshiki Ikeda, Koji Okuwaki, Kaori Fukuzawa, So Nakagawa, Mika Sasaki, Atsuo T. Sasaki, Toshiya Senda

**Affiliations:** 1 Graduate School of Pharmacological Sciences, The University of Tokyo, Japan; 2 Structural Biology Research Center, Institute of Materials Structure Science, High Energy Accelerator Research Organization (KEK), Ibaraki, Japan; 3 Division of Hematology and Oncology, Department of Internal Medicine, University of Cincinnati College of Medicine, OH, USA; 4 Department of Molecular Genetics, Institute of Biomedical Science, Kansai Medical University, Hirakata, Japan; 5 Graduate School of Pharmaceutical Sciences, Osaka University, Japan; 6 Department of Molecular Life Science, Tokai University School of Medicine, Isehara, Japan; 7 Department of Cancer Biology, University of Cincinnati College of Medicine, OH, USA; 8 Department of Neurosurgery, Brain Tumor Center at UC Gardner Neuroscience Institute, Cincinnati, OH, USA; 9 Institute for Advanced Biosciences, Keio University, Yamagata, Japan; 10 Department of Clinical and Molecular Genetics, Hiroshima University Hospital, Japan; 11 Department of Accelerator Science, School of High Energy Accelerator Science, SOKENDAI (The Graduate University for Advanced Studies), Ibaraki, Japan; 12 Faculty of Pure and Applied Sciences, University of Tsukuba, Ibaraki, Japan

**Keywords:** energy metabolism, GTP, GTP sensing kinase, molecular evolution, PI5P4Kβ

## Abstract

Over 4 billion years of evolution, multiple mutations, including nucleotide substitutions, gene and genome duplications and recombination, have established *de novo* genes that translate into proteins with novel properties essential for high-order cellular functions. However, molecular processes through which a protein evolutionarily acquires a novel function are mostly speculative. Recently, we have provided evidence for a potential evolutionary mechanism underlying how, in mammalian cells, phosphatidylinositol 5-phosphate 4-kinase β (PI5P4Kβ) evolved into a GTP sensor from ATP-utilizing kinase. Mechanistically, PI5P4Kβ has acquired the guanine efficient association (GEA) motif by mutating its nucleotide base recognition sequence, enabling the evolutionary transition from an ATP-dependent kinase to a distinct GTP/ATP dual kinase with its *K*_M_ for GTP falling into physiological GTP concentrations—the genesis of GTP sensing activity. Importantly, the GTP sensing activity of PI5P4Kβ is critical for the manifestation of cellular metabolism and tumourigenic activity in the multicellular organism. The combination of structural, biochemical and biophysical analyses used in our study provides a novel framework for analysing how a protein can evolutionarily acquire a novel activity, which potentially introduces a critical function to the cell.

## Introduction

Kinases play critical roles in a wide variety of cellular processes, such as signal transduction, transcription and metabolism, and the human genome contains far over 500 kinases [1]. Even though there is diversity in their structure, substrate specificity and participating pathways, the vast majority of kinases, from bacteria to humans, use ATP as the physiological phosphate donor [2–5]. By contrast, a recent study challenges this dogma and suggests that, in higher animals, the specificity has been disrupted with important functional implications.

Applying a proteome-wide screening of GTP interacting proteins with a follow-up biochemical and the structure-based reverse genetics approach, we showed that the GTP sensing activity of phosphatidylinositol 5-phosphate 4-kinase β (PI5P4Kβ) is critical for metabolic adaptation and tumourigenesis [6,7]. Transformed cells that lack the GTP sensing activity of PI5P4Kβ cannot sustain their growth under xenograft conditions, demonstrating that the GTP sensing function endows a survival advantage. The critical GTP sensing activity role of PI5P4Kβ, especially in cancer cells, establishes this lipid kinase as a target of cancer therapeutics [7]. In line with the notion, increased GTP synthesis is reported in cancer cells [8] and the higher cellular GTP concentration can be a metabolic signature that the GTP sensor kinase can sense. Moreover, the finding that PI5P4Kβ can use GTP more efficiently than ATP reveals this to be the first GTP-utilizing kinase with a biologically relevant GTP-dependent activity.

Interestingly, a molecular evolutionary analysis of phosphoinositide sequences revealed that PI5P4Kβ was derived from an ATP-dependent kinase, PI4P5K [1,9], after the emergence of multicellular organisms [10]. This fact suggests that the GTP sensing activity of PI5P4Kβ can be related to a newly adopted cellular function, which modulates cellular processes of multicellular organisms, presumably through intracellular metabolic communications. In the recent study published in the journal, *Structure*, we uncovered the molecular process by which the PI5P4Kβ was evolved to demonstrate the GTP sensing activity [11]. An integrated structural and biochemical strategy we developed here to analyse the molecular evolution of PI5P4Kβ would provide a new framework to investigate the mechanism by which organisms develop a new protein function. Here, we will briefly summarize the strategy we adopted to analyse the molecular evolution of PI5P4Kβ and describe the importance of nondetrimental *cryptic* mutations, which pave the way for smooth evolutionary transition—leading to the acquisition of new protein activity. We will also discuss the principle of how the new protein activity renders a high-order function to cells.

## The GTP sensing kinase, PI5P4Kβ: Its evolutional origin

PI5P4Kβ, also called the type II phosphatidylinositol 5-phosphate 4-kinase or the type II PIP kinase, is a kinase that phosphorylates PI(5)P to produce PI(4,5) P_2_ (4). While PI5P4Kβ regulates the amount of two lipid second messengers, PI(5)P and PI(4,5)P_2_, it has been suggested that a major role of PI5P4K is to regulate the levels of PI(5)P since the majority of PI(4,5)P_2_ is produced by another pathway by phosphatidylinositol 4-phosphate 5-kinase (PI4P5K) or the type I PIP kinase from PI(4)P [6,9]. The knock-out mice of the PI5P4Kβ display reduced body weight, resistance to obesity induced by high-fat diets and increased insulin sensitivity [12].

Since its identification in 1997 [13], PI5P4Kβ was undoubtedly considered to utilize ATP for its enzymatic reaction. However, our recent study uncovered that PI5P4Kβ is a unique GTP-utilizing kinase that preferably uses GTP over ATP [6,7]. Even in the presence of a physiological concentration of ATP (1–2 mM) [14], more than 50% of PI(4,5)P_2_ was produced by using GTP at 0.5 mM concentration [6]. PI5P4Kβ displayed a higher GTP-dependent kinase activity than ATP, regardless of PI(5)P levels and the types of divalent metal ions [6].

Molecular evolutionary analyses suggested that PI5P4K diverges from PI4P5K at the ancestral lineage of Choanoflagellates and Filasterea [10]. Interestingly, the ancestral PI4P5K is a canonical kinase that preferably utilizes ATP [6,9,15]. The accumulating evidence argues that PI5P4Kβ might have evolved from an ATP-type kinase and adopted a GTP-utilizing activity to function as a GTP sensor at a certain point during evolution. However, it remains elusive how PI5P4Kβ acquires the novel GTP-dependent kinase activity besides the original ATP-dependent activity. To address this central question, we first conducted a comprehensive structural analysis of structurally known kinases and G-proteins [11].

## Comprehensive analysis of ATP and GTP recognition leads to identifying the GEA motif

**Fig. 1.**
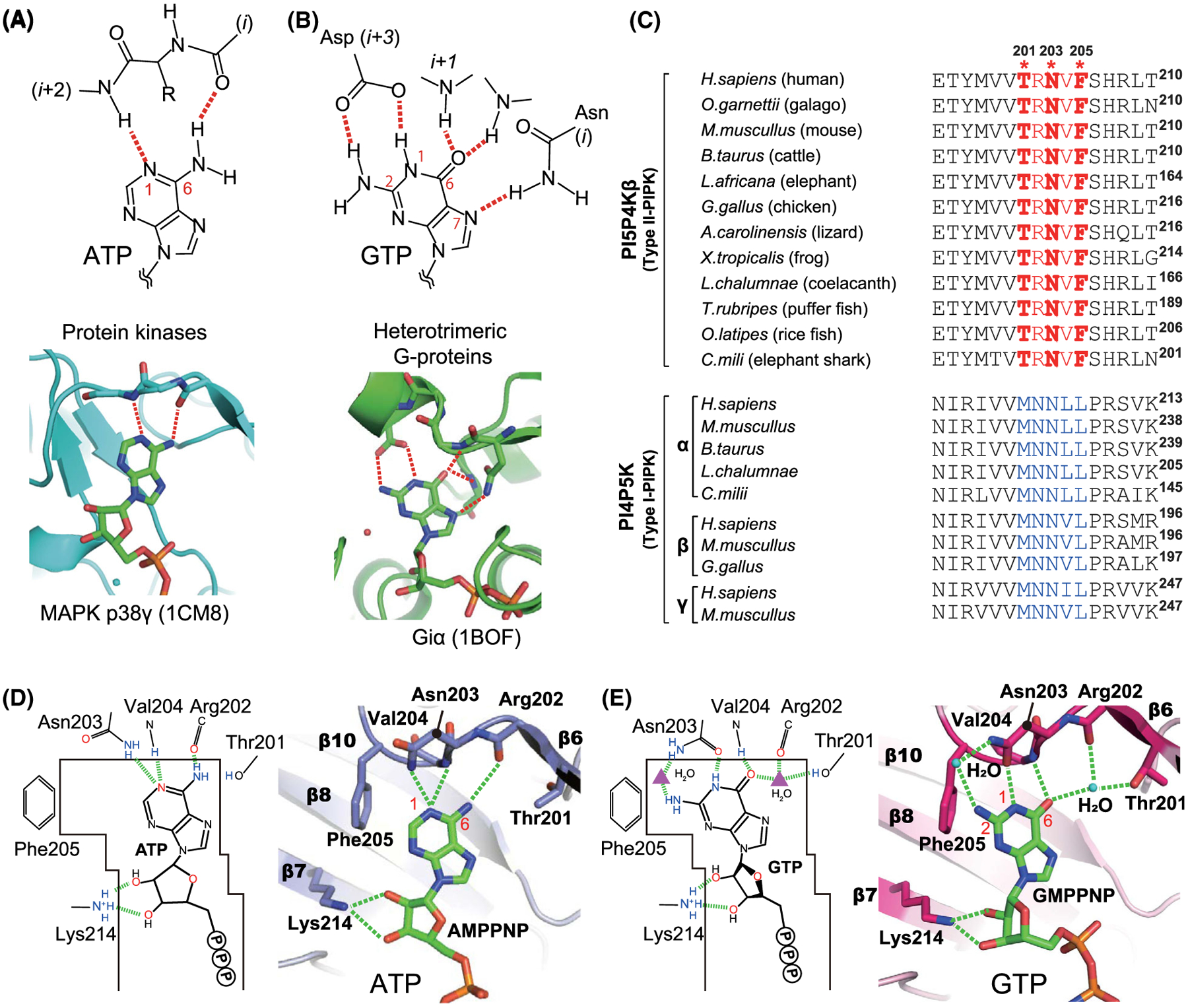
Comparison of nucleotide recognition modes of kinases, G-proteins and PI5P4Kβ. (A and B) Typical nucleotide recognition modes of (A) kinases and (B) G-proteins. (C) Sequence alignment of PI5P4Kβ and PI4P5K family proteins. The GTP-recognizing GEA motif is highlighted in red. For PI4P5K family proteins, the ATP-recognizing MNNψL sequences are coloured in blue. Asterisks indicate the positions Thr-201, Asn-203 and Phe-205 in human PI5P4Kβ. (D and E) Distinct ATP- and GTP-binding modes of human PI5P4Kβ. (A) ATP- and (B) GTP-binding modes of PI5P4Kβ determined in our previous study [1,11]. The hydrogen bonds between purine nucleotide triphosphates (PNTs) and PI5P4Kβ are shown in red dotted lines. PDB ID for the ATP- and GTP-bound structures are 6K4H and 6K4G, respectively. Figure is adapted from [11]. The structure graphics were generated by the PyMOL Molecular Graphics Software (version 2.3.0a0 Open-Source). The multiple sequence alignment was generated by the CLUSTAL.

The ATP recognition by kinases was comprehensively analysed by all protein, PI and IP kinases structures deposited in the protein data bank (PDB) [11]. We identified 968 interactions in these 660 kinase structures and found that the N(1) and NH_2_(6) positions of ATP adenine base typically form two hydrogen bonds between the mainchain amide and the carbonyl groups from the (*i* + 2)^th^ and *i*^th^ residues, respectively (Fig. 1A).

The same strategy was applied to 128 G-protein structures in the PDB. In the G-protein structures, the N(1) and NH_2_(2) of the guanine base are simultaneously recognized by the sidechain carboxylate of Asp in the NKX**D** motif [16,17]. In most cases, the N (7) position forms a hydrogen bond with the Nδ of Asn in the **N**KXD motif. In addition, the O(6) creates a hydrogen bond(s) with a neighbouring *i* + 1^th^ Lys and/or a remote mainchain amide group(s) (Fig. 1B).

Comparison of these ATP/GTP-binding modes with PI5P4Kβ revealed the dual specific nucleotide base recognition _201_TRNVF_205_ sequence (residue number corresponds to that of human; Fig. 1C), which we designate as the GEA (Guanine Efficient Association) motif. The GEA motif binds to ATP similar to other kinases utilizing the mainchain amide group of Val-204 and carbonyl group of Arg-202 to recognize N(1) and NH_2_(6) positions of the adenine base, respectively (Fig. 1D). As for GTP-recognition mode, PI5P4Kβ uses amino acid sidechains to form hydrogen bonds to N(1), NH_2_(2) and O(6) of the guanine ring (Asn-203 for N(1) and NH_2_(2) and Thr-201 for O(6)). However, the interacting residues are distinct from those of the G-protein. Asn-203 in the _201_TR**N**VF_205_ sequence is structurally located at the corresponding position of the conserved Asp residue of the G-protein, and its Oδ and Nδ atoms form direct and indirect hydrogen bonds with N(1) and NH_2_(2), respectively (Fig. 1E). In addition, Thr-201, Arg-202 and Val-204 in the **TR**N**V**F sequence form a hydrogen-bond network including O(6) of the guanine base with a water molecule (Fig. 1E). Such a GTP interaction is enabled by a 1.5 Å shift of the base moiety relative to the ATP. Although the presence of a water molecule in the hydrogen-bond network is unique in GTP recognition, a fragment molecular orbital (FMO) analysis supports its significant contribution to guanine base recognition. Taken together, PI5P4Kβ has a unique dual nucleotide recognition mode utilizing a short GEA motif, which could account for the ideal affinity of PI5P4Kβ to GTP to express GTP sensing function.

## Promiscuous ITP and XTP recognition by the GEA motif

**Fig. 2.**
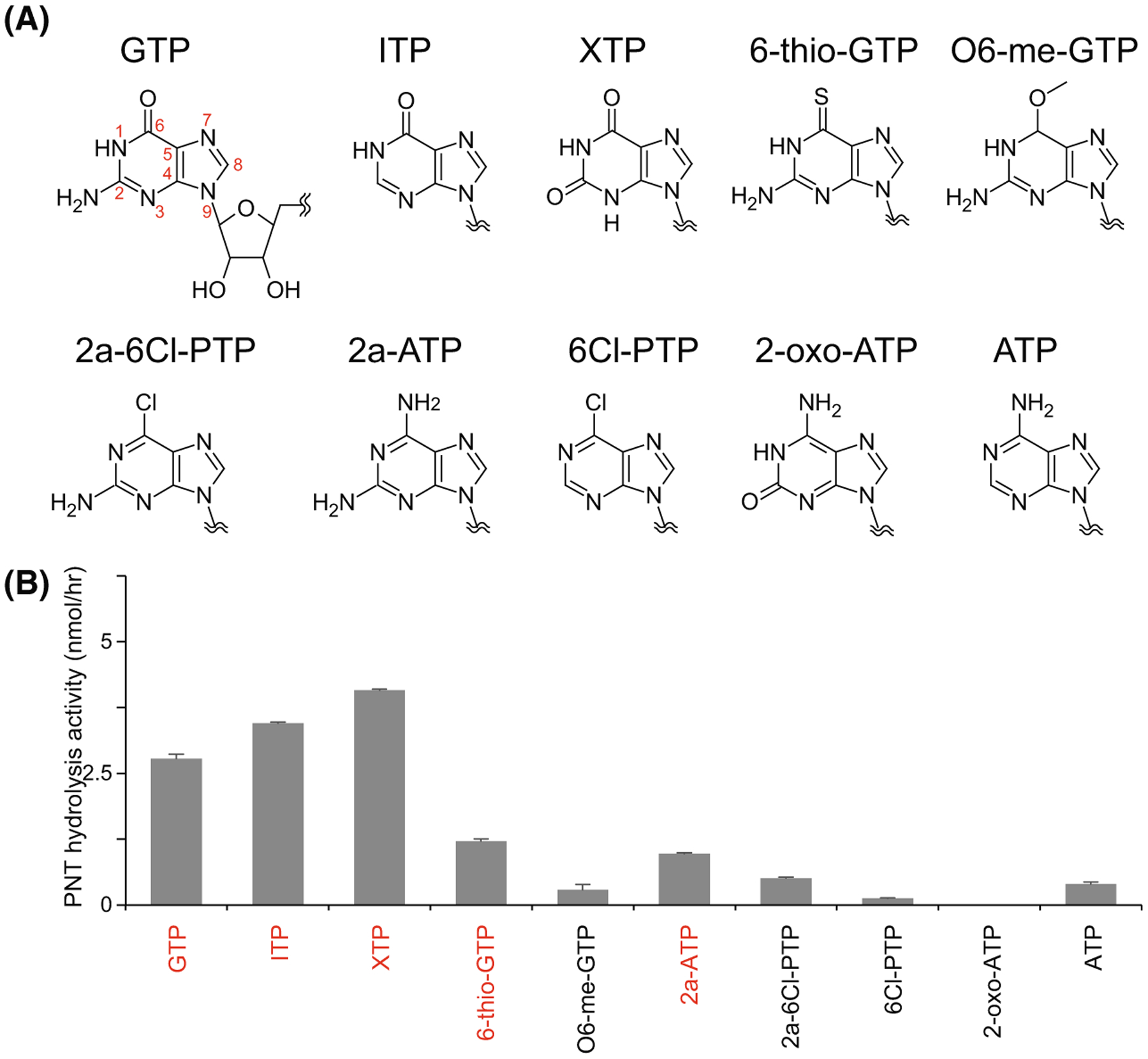
Promiscuous PNT hydrolysis activity of PI5P4Kβ. (A) Chemical structures of ATP and GTP, along with other PNTs used in this study. (B) PNT hydrolysis activity of PI5P4Kβ. 250 μM PNTs were mixed with 2 μM PI5P4Kβ. The average values from three experiments are shown with error bars (standard deviations; S.D.). Highly hydrolyzed nucleotides (>0.1 of the ratios) are indicated in red. Figure is adapted from [11].

To further investigate the nucleotide base interactions via the GEA motif, we exploited the extensive structure and activity of human PI5P4Kβ in complex with 10 PNTs possessing different functional groups or protonation states at the 1^st^, 2^nd^, and 6^th^ positions (Fig. 2A). For this purpose, the hydrolysis activity assay was assayed by NMR. NMR-based hydrolysis assay has been shown to faithfully recapitulate the characteristic GTP preference of the kinase [1]. Unlike radio-labelled PNT methods [6], this NMR-based strategy allows the activity of each nucleotide to be analysed in parallel in the same conditions without introducing any chemical modifications and additional reagents to detect the protein activity [11].

The NMR activity assay revealed that PI5P4Kβ promiscuously hydrolyzed inosine triphosphate (ITP), xanthosine triphosphate (XTP), 6-thio-GTP and 2-amino-ATP (2a-ATP), in addition to GTP and ATP (Fig. 2B). The results indicate that NH_2_(2) is relatively dispensable, while NH(1) and O(6) are required for the binding to PI5P4Kβ. The crystal structures of PI5P4Kβ-ITP, PI5P4Kβ-XTP and PI5P4Kβ−2a-ATP complexes support the structural findings deduced from the NMR analysis [11]. While PI5P4Kβ can utilize at least four nucleotides present in human cells, the negligible cellular concentrations of XTP and ITP relative to ATP and GTP might not affect the ATP/GTP-dependent kinase activity of the WT PI5P4Kβ in living cells.

## Dissecting the evolutionary path toward the GTP sensor

While enzymes are traditionally considered specific to a particular substrate, some can promiscuously utilize other substrates to catalyse different reactions [18,19]. The option of alternative substrates and/or interactions between proteins is in theory a wedge point for functional evolution [18,19]. Since PI5P4Kβ can react with GTP, ITP, XTP and ATP, we focussed on the evolution of the GEA motif of PI5P4Kβ and its substrate specificity.

Among the three amino acid residues that critically contribute to the nucleotide-binding in the GEA motif of PI5P4Kβ, Thr-201 and Phe-205, are substituted to Met and Leu, respectively, in PI4P5K, while Asn-203 is conserved in both PI5P4Kβ and PI4P5K (Fig. 1C). The comparison of genomic sequences and codon usage of 12 diversified vertebrates revealed that, although several nucleotide substitutions occurred at the codons corresponding to the GEA motif in PI5P4Kβ, amino acid sequences encoded by the codon were retained among these species, suggesting the region was under purifying selection [11]. Thus, in the evolutionary analyses, we focussed on the effect of the acquisition of Thr-201 and Phe-205 in the GEA motif in human PI5P4Kβ compared with original Met and Leu, respectively, in PI4P5K, for dissecting the evolution path toward the GTP sensor.

## A trade-off between nucleotide specificity and the GTP-dependent activity in PI5P4Kβ

**Fig. 3.**
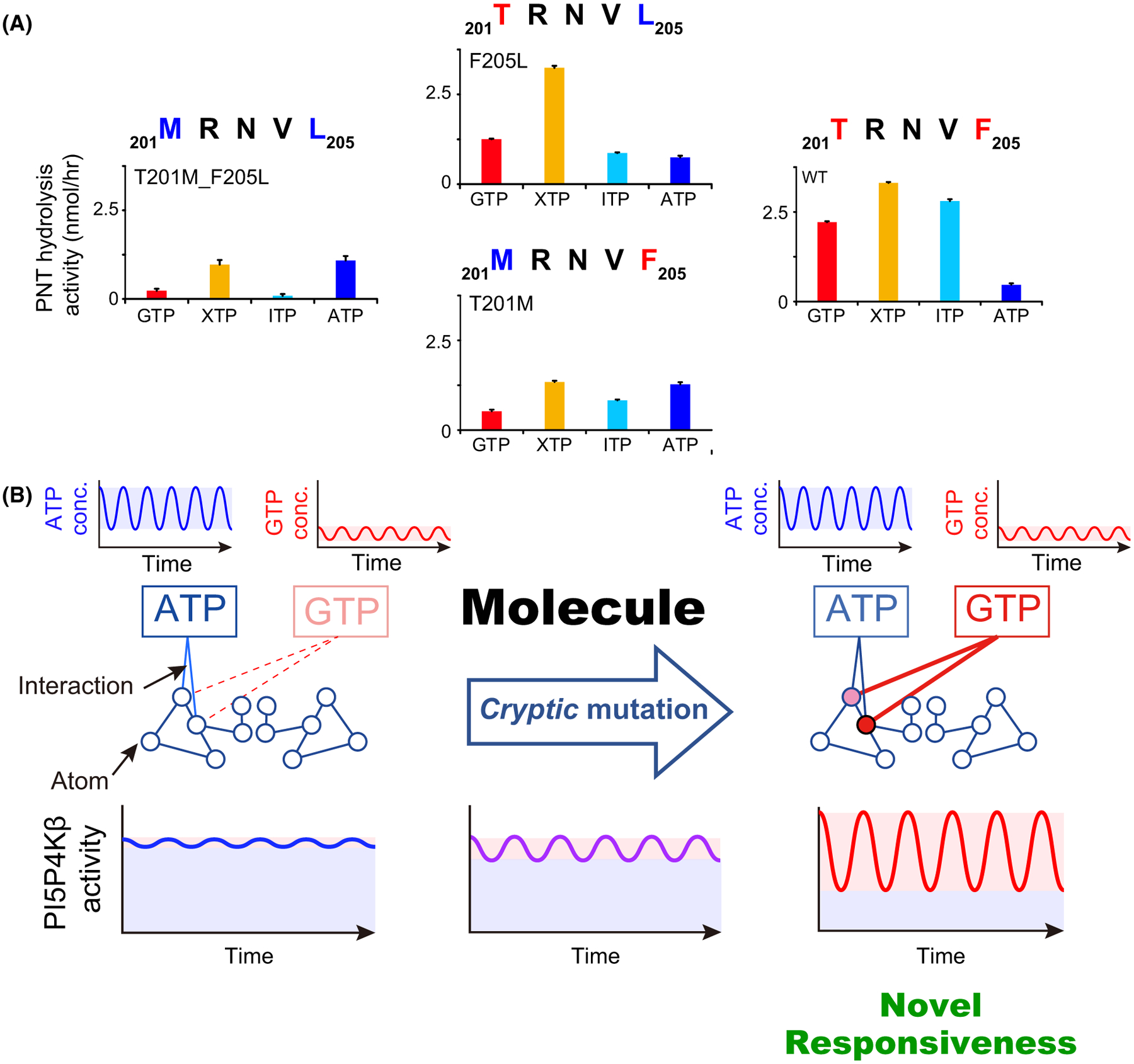
Molecular evolution of the GTP-dependent activity in PI5P4Kβ. (A) PNT hydrolysis activities of the WT PI5P4Kβ and T201M, N203D, N203A, F205L and T201M_F205L double mutants are shown along with the amino acid sequence of the GEA motif. The average values from the three experiments are shown with error bars (S.D.). Figure is modified from [11]. (B) Molecular evolution of the GTP-dependent activity in PI5P4Kβ. Two critical mutations induced the novel interaction with GTP, which endows the novel responsiveness to GTP while largely retaining basal ATP-dependent activity, in evolution pass via the *cryptic* mutations.

The PI5P4Kβ^T201M_F205L^ double mutant, which has the same amino acids as the ancestral ATP-dependent kinase (i.e. PI4P5K), essentially abolished the hydrolysis activity for GTP, ITP and XTP and showed vigorous ATP-hydrolysis activity (Fig. 3A). Thus, the PI5P4Kβ^T201M_F205L^ mutant is reverted from the GTP-dependent kinase to its ancestral ATP-dependent kinase. Intriguingly, the acquisition of Thr at the 201^st^ position (see PI5P4Kβ^F205L^) makes the kinase more active to GTP, while largely retaining the ATP activity. However, the gain in the GTP-hydrolysis activity is associated with a much stronger activity to XTP. The acquisition of Phe at the 205^th^ position (see PI5P4Kβ^T201M^) also did not affect its hydrolysis activity to ATP while slightly enhancing GTP-hydrolysis activity. Again, the hydrolysis activity to XTP and ITP was increased. The strongest GTP-hydrolysis activity was achieved when both Thr-201 and Phe-205 were acquired; however, the resultant WT PI5P4Kβ showed substantial activities to XTP and ITP. Thus, the GTP-hydrolysis activity coincides with extended activity to XTP and ITP, suggesting a trade-off between nucleotide specificity and the GTP-dependent kinase activity in the WT PI5P4Kβ. These results reveal that evolutional acquisition of the GEA motif results in the acquisition of GTP responsivity. It should be noted that we confirmed all mutant proteins retain their original protein folds, by determining their crystal structures [11].

## Role of cryptic mutations in the evolution of GTP sensing function

**Fig. 4.**
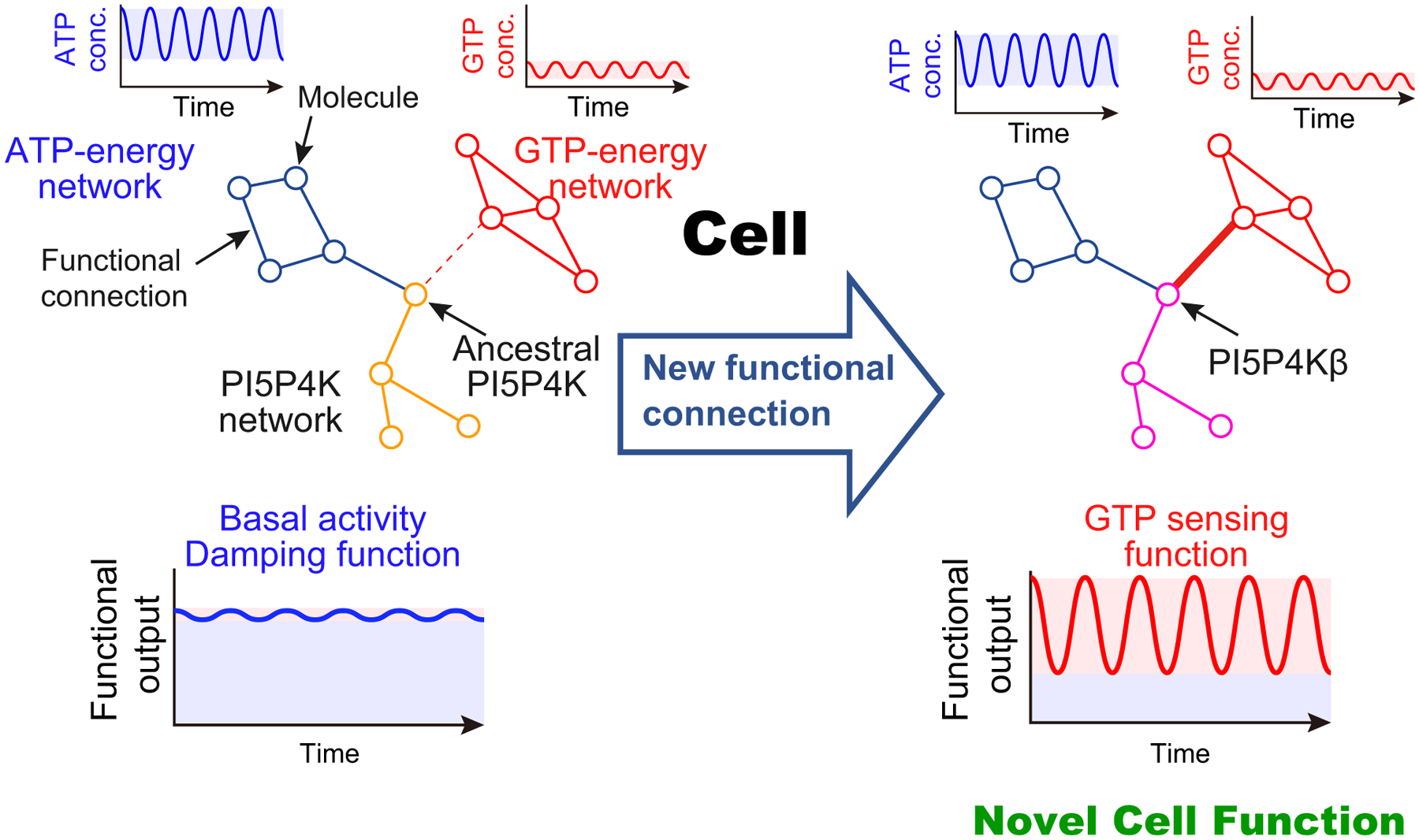
Evolutional acquisition of the GTP sensing function through rewiring of the functional cellular network. (Left) Ancestral PI5P4K only is connected to the ATP-energy network, and fluctuation in the ATP concentration was largely attenuated in the PI5P4K network to show the basal activity. (Right) Two critical mutations introduced the novel functional connection to the GTP-energy network, which allows the endowing of the novel GTP responsiveness to the PI5P4K network and a novel function to the cell.

The evolutional analysis of the GEA motif unveiled that the gain in GTP-dependent kinase activity was achieved by loss of nucleotide specificity. This specificity change can be reconsidered in a cellular context where these nucleotides coexist in variable concentration ranges [14]. Substantial ATP-dependent activity is observed with PI5P4Kβ (Fig. 3A). Thus, considering the higher cellular concentrations of ATP than GTP [14], half of the PI5P4Kβ activity at most would originate from ATP at physiological nucleotide concentrations. By contrast, since the intracellular concentrations of ITP and XTP are negligibly low, the extended activity of XTP and ITP would not contribute to the biological function of PI5P4Kβ. In addition, since the concentration of ATP (1–5 mM) is much higher than the *K*_m_ value of PI5P4Kβ for ATP (238 μM), the enzymatic activity originating from ATP is mostly insensitive to the physiological variations of ATP concentrations providing the ‘*basal*’ activity of PI5P4Kβ (Fig. 3B, blue shadow). However, the role of the ‘basal’ ATP-dependent activity of PI5P4Kβ is unclear as it can work as a damper that attenuates the small fluctuation in nucleotide concentrations, including those of ITP and XTP, and provides stability to the energy homeostasis system (Fig. 4, left).

On the other hand, the two mutations in PI5P4Kβ primarily change the responsiveness of the protein to the ambient cellular GTP concentration (Fig. 3B, red shadow). This leads to the establishment of an additional functional connection between GTP-energy homeostasis and the phosphoinositide signalling system in the cell (Fig. 4, right). Given the importance of the GTP concentration in controlling energy metabolism and proliferation under stress conditions [6], establishing a new mode of signalling that utilizes GTP likely introduces a high-order cellular function to the cell.

Toward this end, it would be interesting to examine in detail the fate of the original ATP-dependent activity in the context of evolutionary pressures. Neither PI5P4Kβ^T201M^ nor PI5P4Kβ^F205L^ loses its original ATP-dependent activity (Fig. 3A). Therefore, having the *semi-optimal* sequence (_201_TRNVL_205_ or _201_MRNVF_205_), which might exist in the evolutional path toward the GTP-recognizing _201_TRNVF_205_ sequence, is not detrimental to the protein as it would retain its ancestral basal ATP-dependent activity (Fig. 3B). Such functionally nondetrimental *cryptic mutations* might allow a smooth evolutional transition of an ATP-dependent kinase to a GTP sensing kinase and have less impact on the cellular functions. This notion would be supported by the fact that we have reported on establishing a cell line that only has PI5P4Kβ^F205L^ [6]. Species with a single mutation in the GEA motif has not been identified to our knowledge. However, considering the probability of two mutations occurring simultaneously at different sites would be much rarer than having two mutations separately. We speculate that the period that the species with a single *cryptic* mutation in the GEA motif would exist as the bridge toward the fully functional GTP sensor.

## How would protein evolution be integrated into a new cellular function?

Taken together, the comprehensive biochemical and structural analysis of PI5P4Kβ uncovers the clear evolutionary path from ATP-dependent kinase to the ATP/GTP dual kinase with GTP sensing activity. The results illuminate how a new biological function could evolve by accumulating *cryptic* mutations, which are already present in genome sequences but without an immediate impact until other conditions come into play (Figs. 3B and 4). These ‘silent’ mutations without positive or negative impact and in that sense initially ‘*cryptic*’ only reveal themselves when an additional function for the cell evolves (Fig. 4, right). Since the functional cellular networks are already compartmentalized in a cell, a single new connection, as in the case of PI5P4Kβ, added to the cell would be readily functional leading to a substantial impact on the whole cellular system. Thus, small changes in substrate specificity could rewire the functional cellular network and could serve to efficiently introduce an entirely new set of high order functionally to the cell (Fig. 4). In this sense, the *cryptic* mutations that accumulate in a way that is functionally near *neutral* would represent a comparatively safe evolution path with extraordinary functional potential. Although in our example of PI5P4Kβ the signalling triggered by the change of GTP concentration is not fully elucidated, the notion of the functional connection between GTP sensing and phosphoinositide signalling strongly suggests this is a legitimate functional mechanism.

Our view of molecular evolution of the GTP sensing kinase is consistent with the observation that a protein with a new function is not necessarily constructed from *de novo* but is co-opted to existing ones by exaptation. Such an evolution is often associated with gene duplications, which provide additional room for yielding novel functions besides the original one [18,19]. This appears to be operative in the case of the evolution of PI5P4Kβ. In the ancestorial lineage, there was only one isotype of PI5P4K, until three isotypes (α, β, and γ) of PI5P4K appeared in vertebrates by two successive gene duplications. It should be noted that the other isotypes of PI5P4K, PI5P4Kα and PI5P4Kγ, have unqualified features as a GTP sensor, although they share high overall sequence similarity and can use GTP as phosphodonor [6,20]. It would also be interesting to determine the nucleotide specificity of the PI5P4Ks before vertebrates (e.g. insects and molluscs).

It should be noted that PI5P4Kβ is not only a promiscuous enzyme but also a ‘moonlighting’ protein [21,22], as it has been shown that PI5P4Kβ may act as scaffolds to recruit PI5P4Kα by heterodimerization [23] and the evolution of the moonlighting activity need to be clarified along with its high-order role in cellular functions.

## A multidiscipline approach to unveil novel protein functions

The co-option of a new activity often occurs when the very weak corresponding activity already coexists with the original activity, as exemplified by PI5P4Kβ here. Other cases are reported in line with this notion [18,19]; however, such a promiscuous activity is often difficult to predict as those functions will not be performed by well-known protein motifs. Therefore, additional mechanistic and structural studies would be needed to improve our understanding and predictability of such a function. Although most of the studies of the protein activity, function and structure–activity relationships are conducted in a way that focusses on a known activity with a few particular substrates and a couple of complex structures, an exhaustive search for the possible substrates with a certain function of interest would be needed to discover a protein with a new function. Thus, a thorough study of its promiscuity with the physiological concentrations in mind would also be essential to unveil the way that the protein function in certain cellular conditions. In addition, extensive protein structural-activity relationship studies with those substrates should be conducted along with the quantum mechanic’s simulation for unveiling the origin of the promiscuity. The accumulation of the data describing such interaction in a quantitative manner might allow us to predict the possible range of substrates for a protein of interest, through machine learning processes. Furthermore, a strategic mutational study to track the evolution path would be needed to understand how the protein has evolved to acquire the novel function.

In this sense, our strategy in discovering a GTP sensing kinase, PI5P4Kβ and the follow-up studies on its promiscuity and evolution represents an integrated multidiscipline approach to unveil the way that a protein could evolve to express not only new biochemical but also novel biological functions to adapt environments. The difficulty lies in establishing a method to account for the whole range of different substrates and reactions. In the case of PI5P4Kβ, NMR has shown its ability to observe the reaction with multiple substrates in parallel. Although only 10 analogues were tested in our study, the capability of NMR to separately detect >100 metabolites and their reactions in a mixture without additional chemical modifications would provide a robust way to define the range of promiscuity of an enzyme. The cross-disciplinary approach used in this study offers a novel framework for analysing how proteins acquire a new function in evolution that renders a high-order cellular function, which might provide a survival advantage.

## Data Availability

The structural data that support the findings of this study are openly available in Protein Data Bank: WT-GMPPNP complex, 6K4G; WT-AMPPNP complex, 6K4H. Other data are available from the corresponding authors upon reasonable request (kohtakeuchi@mol.f.u-tokyo.ac.jp).

## Abbreviations

2a-ATP: 2-amino-ATP
FMO: fragment molecular orbital
GEA: guanine efficient association
ITP: inosine triphosphate
PDB: protein data bank
PI(4,5)P_2_: phosphatidylinositol 4,5-bisphosphate
PI(5)P: phosphatidylinositol 5-phosphate
PI4P5K: phosphatidylinositol 4-phosphate 5-kinase
PI5P4Kβ: phosphatidylinositol 5-phosphate 4-kinase β
PNTs: purine nucleotide triphosphates
XTP: xanthosine triphosphate
